# A novel homozygous missense *SLC25A20* mutation in three CACT-deficient patients: clinical and autopsy data

**DOI:** 10.1038/s41439-020-0098-y

**Published:** 2020-04-16

**Authors:** Yasutsugu Chinen, Kumiko Yanagi, Sadao Nakamura, Noriko Nakayama, Motoko Kamiya, Mami Nakayashiro, Tadashi Kaname, Kenji Naritomi, Koichi Nakanishi

**Affiliations:** 1 Department of Child Health and Welfare (Pediatrics), Graduate School of Medicine, University of the Ryukyus, Nishihara, Okinawa Japan; 2 Genetic Counseling Room, University of the Ryukyus Hospital, Nishihara, Okinawa Japan; 3 Department of Genome Medicine, National Center for Child Health and Development, Tokyo, Japan; 4 Department of Pediatrics, Naha City Hospital, Naha, Okinawa Japan; 5 Department of Pediatrics, Okinawa Prefectural Nanbu Medical Center Children’s Medical Center, Haebaru, Okinawa Japan; 6 Okinawa Nanbu Habilitation and Medical Center, Naha, Japan; 7 Present Address: Center for Medical Genetics, Shinshu University Hospital, Matsumoto, Japan

**Keywords:** Disease genetics, Medical genetics

## Abstract

Carnitine-acylcarnitine translocase (CACT) deficiency is a fatty acid ß-oxidation disorder of the carnitine shuttle in mitochondria, with a high mortality rate in childhood. We evaluated three patients, including two siblings, with neonatal-onset CACT deficiency and revealed identical homozygous missense mutations of p.Arg275Gln within the *SLC25A20* gene. One patient died from hypoglycemia and arrhythmia at 26 months; his pathological autopsy revealed increased and enlarged mitochondria in the heart but not in the liver.

Mitochondrial fatty acid ß-oxidation has a pivotal role in energy production during starvation, particularly in the heart and skeletal muscle, because they depend mainly on the oxidation of long-chain fatty acids. The metabolism of cytosolic long-chain fatty acids into acyl-CoA occurs in the intramitochondrial space via the carnitine shuttle, which consists of carnitine palmitoyltransferase (CPT) I, CPT II, and carnitine-acylcarnitine translocase (CACT)^1–3^. High levels of CACT mRNA transcripts, encoded by the *SLC25A20* gene on chromosome 3p21 (MIM*613698), are present in the heart, skeletal muscle, and liver; lower levels are present in the brain, kidney, pancreas, and lung^4,5^. CACT deficiency (MIM212138) was first described in 1992^6^; it is a rare autosomal-recessive disease of mitochondrial fatty acid oxidation with a high mortality rate (65%). In CACT deficiency, abnormal acylcarnitine profiles revealed high levels of C16-carnitine and (C16 + C18:1)/C2, including C14/C3, similar to those found in CPT II deficiency in a mass screening of newborns^7^. Most patients (82%) with neonatal-onset CACT deficiency have hypoketotic hypoglycemia, hyperammonemia, skeletal muscle weakness, and cardiomyopathy with arrhythmia, leading to cardiac arrest^8^. To prevent adipose tissue lipolysis, sufficient intake of glucose to maintain normal plasma glucose levels, the intake of medium-chain triglycerides (MCTs), and a restriction of long-chain fatty acid intake is required. Brivet (2004) recommended that L-carnitine therapy be administered intravenously, with intravenous injection of high concentrations of glucose in emergencies and orally 3–4 times/day when the patient is stable^9^. Despite oral carnitine supplementation, plasma carnitine levels remain very low in CACT-deficient patients.

At the Pediatric Department of the University of the Ryukyus, three patients in two unrelated families (Fig. 1: Family-a, -b) from Okinawa, Japan, with neonatal onset of CACT deficiency diagnosed by metabolite and genomic mutation analyses, were followed up from February 2008 to October 2017. After direct Sanger sequencing for all exons of *SLC25A20*, a homozygous missense variant of NM_000387.6:c.824G>A (p.Arg275Gln) in the *SLC25A20* gene (Fig. 2a) was identified in the three patients [**P1**, **P2**, **P3**], included two siblings [**P2**, **P3**]. The affected amino acid is evolutionarily conserved from fungi to humans, emphasizing that the residue is likely to be functionally important. This was predicted to be a disease-causing mutation by PolyPhen-2 software (http://genetics.bwh.harvard.edu/pph/) and Mutation Taster (http://www.mutationtaster.org), with normal activity of the CPT II enzyme retained [**P1**]. Although we could not find this variant in ClinVar (https://www.ncbi.nlm.nih.gov/clinvar/) and the Japanese genome database of Human Genetic Variation (http://www.hgvd.genome.med.kyoto-u.ac.jp), we found it in gnomAD (https://gnomad.broadinstitute.org). The frequency of this variant is very low: 0.00002784 [allele count: 7/251396, east Asian: 1/18394, European (non-Finnish): 6/113684)] and has no number of homozygotes. Biochemical analysis of **P1**, **P2**, and **P3** demonstrated high levels of C16 acylcarnitine (14.55, 2.61, 8.61; cut-off > 2.1 [nmol/ml]) and high ratios of C14 to C3 (6.06, 1.03, 1.55; cut-off > 0.408) and (C16 + C18:1) to C2 (3.70, 1.83, 0.79; cut-off > 0.41), as measured in dried blood spots by tandem mass spectrometry.

**Fig. 1:**
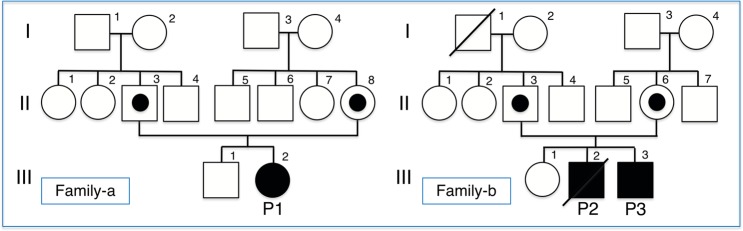
Two family pedigrees with results of the analysis of the *SLC25A20* gene. To our knowledge, there is no apparent consanguinity between the two families. p.Arg275Gln: Family-a; II-3 and II-8 (heterozygosity), III-2 (homozygosity), Family-b; II-3 and II-6 (heterozygosity), III-2 and III-3 (homozygosity).

**Fig. 2:**
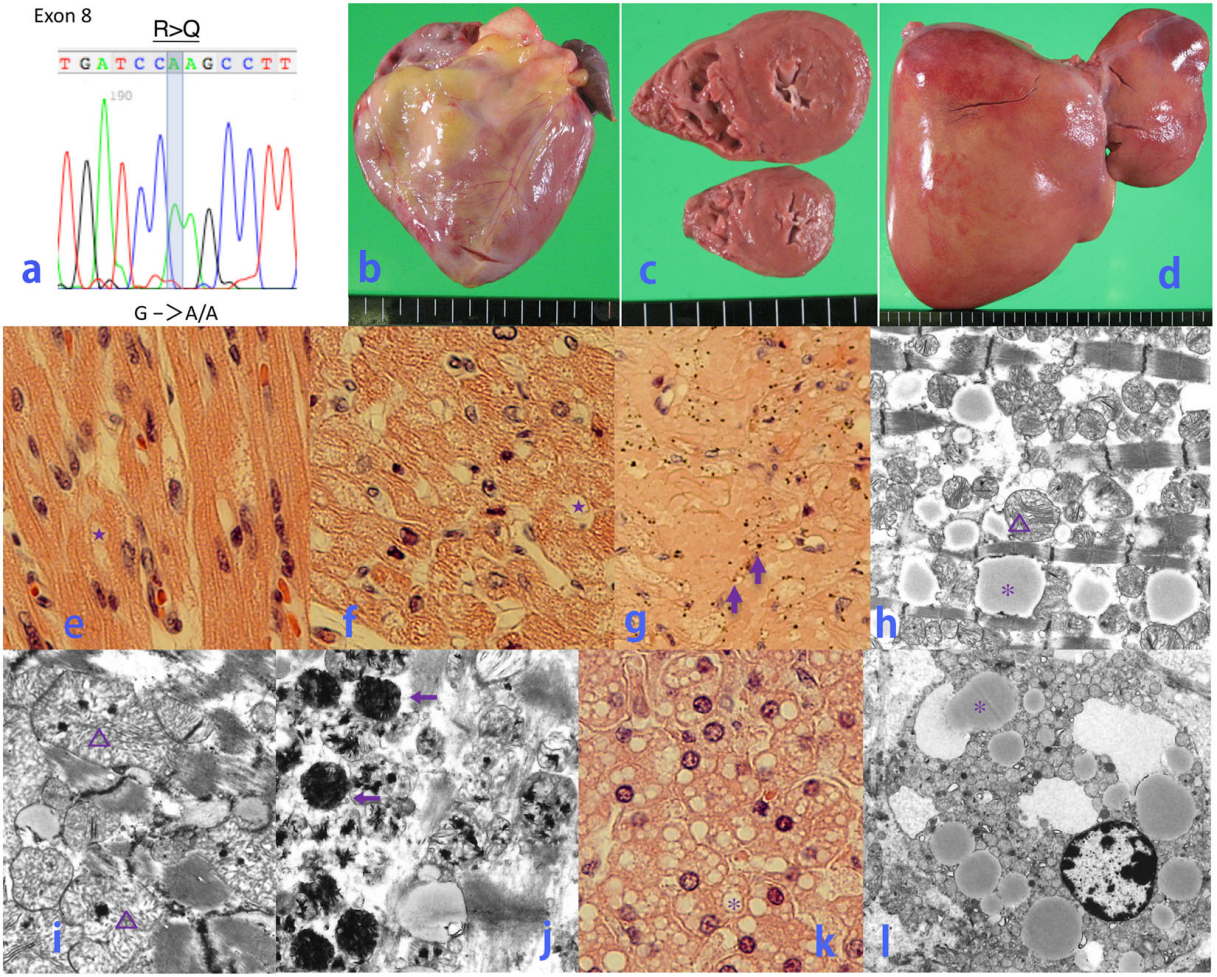
Postmortem pathological imaging and the analysis of the *SLC25A20* gene of P2 at 26 months of age. **a** Molecular analysis of the *SLC25A20* gene revealed homozygous missense mutations of p.Arg275Gln. **b**, **c**, **e**–**j** Heart—mild myocardial thickening, weight 70 g (+0.5 SD). **b** frontal view; **c** transverse ventricle; **e** hematoxylin–eosin (HE) stain ×400; **f** HE stain ×100, partial vacuolation (star), hypertrophy, and eosinophilic changes in cardiomyocytes; **g** HE stain ×200, the deposition of small dark-brown granules (↑); **h**–**j** electron microscopic imaging (EMI) showing small lipid droplets (*), increased numbers of mitochondria, the appearance of giant mitochondria (open triangle), and the deposition of amorphous materials with high electron density (←) in mitochondria. **h** ×2300; **i** ×6900; **j** ×11,500. **d**, **k**, **l** Liver—hepatic steatosis, weight 360 g (−1.0 SD). **d** light yellow liver, frontal view; **k** HE stain ×200, diffuse deposition of small- to medium-sized lipid droplets (*) in liver cells; **l** EMI ×2300, the deposition of small lipid droplets (*) in all hepatocytes, no changes in the liver mitochondria. Scale = 0.5 cm (**a**, **b**, **d**).

**P1** was born at 34 weeks and 6 days of gestation with a birth weight of 1844 g (−1.3 SD). She was transferred to a neonatal intensive care unit for observation. At 2 days of age, she suffered from tachypnea, bradycardia, and hyperammonemia (>1000 µg/dl) [normal range: 36–86 μg/dl]. She was treated with continuous arteriovenous hemodialysis and L-carnitine and recovered, but she experienced intracranial bleeding. She then received L-carnitine supplementation and was fed a fat-restricted diet. She was hospitalized many times because of infection. At 5 years of age, magnetic resonance imaging (MRI) of her brain demonstrated no abnormal findings, and an echocardiogram revealed no hypertrophic cardiomyopathy. Developmental assessment by the Kyodai-shiki schedule (Kyoto International Social Welfare Exchange Center) identified mild mental retardation (developmental quotient [DQ] = 75) at 4 years and 7 months of age; at 10 years and 8 months, her DQ was 63, her height was 137.4 cm (−0.6 SD), and her weight was 30.8 kg (−0.7 SD).

**P2** was born at 39 weeks of gestation with a birth weight of 2570 g (−1.0 SD). At 2 days of age, he presented with grunting, hypoxemia, hypoglycemia (<40 mg/dl), arrhythmia (wide QRS, ventricular tachycardia), and hyperammonemia (300–400 µg/dl). He recovered after the administration of high concentrations of glucose and an intravenous L-carnitine infusion. At 5 days of age, aspirin was administered into the portal vein for thrombosis. This was followed by L-carnitine supplementation, MCT milk, and a fat-restricted diet. At 26 months of age, after playing on the beach, he presented with a tonic-clonic seizure for 20 min, hypoglycemia, and arrhythmia (wide QRS). He was transferred to our hospital but died of cardiac arrest despite the administration of 20% glucose infusion, epinephrine, and L-carnitine. Postmortem examination showed mild myocardial thickening and hepatic steatosis (Fig. 2b–d, k). His heart weight was 70 g (+0.5 SD) [64.9 ± 10.05 g at 2 years of age], liver weight was 360 g (−1.0 SD) [463.81 ± 103.51 g at 2 years of age], and the width of the left ventricle wall was 0.97 mm (+1.8 SD) [0.70 ± 0.15 mm at 2 years of age]^10^. The postmortem pathological findings of **P2** showed partial vacuolation, hypertrophy, and eosinophilic changes in cardiomyocytes (Fig. 2e–f), with small dark-brown granules being found in some areas (Fig. 2g). No invasion of inflammatory cells or myocardial necrosis was observed. Electron microscopic imaging (EMI) of cardiomyocytes showed the presence of small lipid droplets, increased numbers of mitochondria, the appearance of giant mitochondria, and the deposition of amorphous materials with high electron density in mitochondria (Fig. 2h–j). The diffuse deposition of small- to medium-sized lipid droplets in liver cells was notable (Fig. 2k), with small lipid droplets being observed in all hepatocytes (Fig. 2l). Ambiguous vacuoles were also frequently present in hepatocytes. In contrast to the myocardial mitochondria (Fig. 2h–j), there were no changes observed in liver mitochondria (Fig. 2l).

**P3**, a younger brother of **P2**, was born at 38 weeks of gestation with a birth weight of 2676 g (−0.7 SD), length of 47.8 cm (−0.5 SD), and head circumference of 32 cm (−0.7 SD). He had good activity at birth; however, 30 min after birth, he had hypoglycemia (24 mg/dl) [normal range: 50–120], and he was treated with 10% glucose. Five hours after birth, he had apnea and was treated with an intravenous infusion of L-carnitine (100 mg/kg/day) and MCT milk (formula 721, Meiji Dairies Corporation, Tokyo, Japan). At 7 months of age, he failed to thrive and received tube feeding. At 11 months of age, an echocardiogram revealed hypertrophic cardiomyopathy. His left ventricular ejection fraction (LVEF) was 35% (normal range > 55%), and his level of N-terminal pro-B-type natriuretic peptide (NT-proBNP, a cardiac biomarker of heart failure and cardiomyopathy^11,12^) was 2176 pg/ml (normal range: 0.0–54.9). In the hospital, an intravenous infusion of L-carnitine (100 mg/kg/day) was continuously administered for 14 days. Subsequently, his level of NT-proBNP decreased from 2897 to 599.4 pg/ml, and LVEF increased to 44.8%. At 12 months of age, **P3** could walk alone; at 13 months of age, he had a gastrostomy for tube feeding. At 4 years and 1 month of age, an echocardiogram and chest X-ray demonstrated dilated cardiomyopathy (LVEF 63%, left ventricular internal dimension in diastole (LVIDd) 50.5 mm [z score 3.12], CTR 58%, trivial mitral regurgitation). At 4 years and 5 months of age, the Kyodai-shiki developmental schedule identified mild mental retardation (DQ = 57). At 5 years of age, magnetic resonance imaging of his brain presented no abnormal findings. At 5 years and 6 months of age, his height was 112.4 cm (+0.5 SD), and his weight was 24.1 kg (+1.7 SD). He took simple meals orally, in combination with tube feeding.

In **P1** and **P2**, metabolic decompensation with hyperammonemia in the neonatal period was improved by the administration of high concentrations of glucose and L-carnitine; in **P3**, cardiomyopathy was ameliorated by continuous intravenous infusion of L-carnitine in the hospital at 7 months of age. Our report suggests that L-carnitine might improve cardiac conditions and the general condition under conditions of normal blood glucose levels, as has been previously stated^9,13,14^.

When the hypoglycemia of **P2** did not improve, the administration of L-carnitine was ineffective. **P2** died of hypoglycemia and arrhythmia, and his autopsy demonstrated that mitochondria in the heart were increased and enlarged, whereas they were unchanged in the liver. When cytochrome c or ATP synthase is insufficient, mitochondria compensate by increasing in size and number^15–17^. Because there were no fatal pathological changes—such as myocardial necrosis—observed in the autopsy of **P2**, **P2** might have had a fatal arrhythmia. Specifically, insufficient ATP production, or mitochondrial dysfunction due to an imbalance in the acyl-CoA:CoA ratio, along with the increased production of long-chain fatty acids caused by hypoglycemia, might have led to changes in the electrical activity of myocardial cells. To our knowledge, this is the first description of autopsy findings of CACT deficiency. As there are currently insufficient clinical and fundamental data regarding the mechanism of lethal arrhythmia in CACT deficiency, further investigations are warranted.

## Data Availability

The relevant data from this Data Report are hosted at the Human Genome Variation Database at 10.6084/m9.figshare.hgv.2841.
